# VEGF signaling mediates bladder neuroplasticity and inflammation in response to BCG

**DOI:** 10.1186/1472-6793-11-16

**Published:** 2011-11-07

**Authors:** Marcia R Saban, Carole A Davis, Antonio Avelino, Francisco Cruz, Julie Maier, Dale E Bjorling, Thomas J Sferra, Robert E Hurst, Ricardo Saban

**Affiliations:** 1 Department of Physiology, The University of Oklahoma Health Sciences Center, Oklahoma City, OK 73104, USA; 2 Department of Experimental Biology, Faculty of Medicine of Porto and IBMC, 4200-319 Porto, Portugal; 3 Department of Urology, Hospital São João, Faculty of Medicine of Porto and IBMC, 4200-076 Porto, Portugal; 4 Oklahoma Medical Research Foundation (OMRF), Imaging Core Facility, Oklahoma City, Oklahoma 73104, USA; 5 Department of Surgical Sciences, School of Veterinary Medicine, University of Wisconsin-Madison, Madison, WI 53706, USA; 6 Departments of Pediatrics and Biochemistry and Molecular Biology, University of Oklahoma Health Sciences Center, Oklahoma City, Oklahoma 73104, USA; 7 Departments of Urology, Biochemistry, and Molecular Biology, University of Oklahoma Health Sciences Center, Oklahoma City, Oklahoma 73104, USA

## Abstract

**Background:**

This work tests the hypothesis that increased levels of vascular endothelial growth factor (VEGF) observed during bladder inflammation modulates nerve plasticity.

**Methods:**

Chronic inflammation was induced by intravesical instillations of Bacillus Calmette-Guérin (BCG) into the urinary bladder and the density of nerves expressing the transient receptor potential vanilloid subfamily 1 (TRPV1) or pan-neuronal marker PGP9.5 was used to quantify alterations in peripheral nerve plasticity. Some mice were treated with B20, a VEGF neutralizing antibody to reduce the participation of VEGF. Additional mice were treated systemically with antibodies engineered to specifically block the binding of VEGF to NRP1 (anti-NRP1^B^) and NRP2 (NRP2^B^), or the binding of semaphorins to NRP1 (anti-NRP1 ^A^) to diminish activity of axon guidance molecules such as neuropilins (NRPs) and semaphorins (SEMAs). To confirm that VEGF is capable of inducing inflammation and neuronal plasticity, another group of mice was instilled with recombinant VEGF_165 _or VEGF_121 _into the urinary bladder.

**Results:**

The major finding of this work was that chronic BCG instillation resulted in inflammation and an overwhelming increase in both PGP9.5 and TRPV1 immunoreactivity, primarily in the sub-urothelium of the urinary bladder. Treatment of mice with anti-VEGF neutralizing antibody (B20) abolished the effect of BCG on inflammation and nerve density.

NRP1^A ^and NRP1^B ^antibodies, known to reduce BCG-induced inflammation, failed to block BCG-induced increase in nerve fibers. However, the NRP2^B ^antibody dramatically potentiated the effects of BCG in increasing PGP9.5-, TRPV1-, substance P (SP)-, and calcitonin gene-related peptide (CGRP)-immunoreactivity (IR). Finally, instillation of VEGF_121 _or VEGF_165 _into the mouse bladder recapitulated the effects of BCG and resulted in a significant inflammation and increase in nerve density.

**Conclusions:**

For the first time, evidence is being presented supporting that chronic BCG instillation into the mouse bladder promotes a significant increase in peripheral nerve density that was mimicked by VEGF instillation. Effects of BCG were abolished by pre-treatment with neutralizing VEGF antibody. The present results implicate the VEGF pathway as a key modulator of inflammation and nerve plasticity, introduces a new animal model for investigation of VEGF-induced nerve plasticity, and suggests putative mechanisms underlying this phenomenon.

## Background

It is highly likely that sensory dysfunction is involved in various disorders of the lower urinary tract (LUT) including neurogenic bladder, outflow obstruction, idiopathic detrusor instability, overactive bladder, painful bladder syndrome, and diabetic neuropathy involving the bladder. In addition, chronic pathological conditions that cause tissue irritation or inflammation can alter the properties of sensory pathways, leading to a reduction in pain threshold and/or an amplification of painful sensation (hyperalgesia) [1]. Depending on the pathology, several mediators and their respective receptors have been proposed to modulate peripheral nerve plasticity in the LUT, including but not limited to: purinergic receptors in general [2] or P2X receptor in particular [3], transient receptor potential vanilloid subfamily 1 (TRPV1) [1], substance P acting on NK1 receptors [4], protease activated receptors [5], and nerve growth factor and its receptors [6].

The new hypothesis being tested in this manuscript is that increased levels of VEGF observed during bladder inflammation provoke nerve plasticity. This hypothesis is based on evidence indicating that nerves and blood vessels are associated, follow a common molecular pathway during development, and key molecules responsible for their development may continue to control their plasticity in adulthood [7]. The finding that mutant mice (neurogenin1/neurogenin2 double knockout embryos) lacking sensory nerves also present disorganized blood vessel branching [8], suggests that local signals such as VEGF supplied by nerve fibers, may provide a cue that determines blood vessel patterning. In contrast, administration of VEGF can support and enhance the growth of regenerating nerve fibers, probably through a combination of angiogenic, neurotrophic, and neuroprotective effects [9].

In this context, many proteins that were originally discovered to be required for axon guidance have recently been implicated in the development of the vascular [10] and lymphatic systems [11]. Perhaps the most striking observation is that angiogenic factors, when deregulated, contribute to various neurological disorders, such as neurodegeneration. The prototypic example of this cross-talk between nerves and vessels is the vascular endothelial growth factor, VEGF [12]. Although originally described as a key angiogenic factor, it is now well established that VEGF also plays a crucial role in development of the nervous system [12].

Among the neuronal guidance molecules, neuropilins (NRPs) and plexins, and their ligands, semaphorins and VEGF have been extensively studied in the central nervous system. They represent large families of molecules that can transduce signals essential for the regulation of neuronal repulsion and attraction, cell shape, motility, and cell-cell interactions [13-15].

Plexins are similar to the Toll-like receptors (TLRs) in their evolutionary conservation from flies to mammals. In particular, plexin A4 has been shown to be required for bacteria and LPS to engage TLR and trigger the downstream signal transduction pathway including activation of Rac1, c-Jun N-terminal kinase, NF-kB and AP-1 [16]. In addition, plexin-A4 in macrophages is required for optimal cytokine production, including TNFα and IL-6, upon bacterial challenge [16].

NRPs are transmembrane glycoproteins that were initially identified as co-receptors for plexin that mediate the effects of class-3 semaphorins on axon guidance [17]. NRP-1 has high affinity for Sema-3A, whilst NRP-2 homodimers have high affinity for Sema-3F [18]. The diversity of function of these guidance molecules resides in their capacity to also function as co-receptors for VEGF enhancing its binding to VEGF receptors [19]. It has become clear that in the adult organism, NRPs participate in many processes, such as angiogenesis and immune response [20]. NRP1 is associated with blood vessel development, whereas NRP2 was initially identified as a semaphorin receptor, and mediator of axon guidance [17] and lymphatic vessel development [21].

Of relevance to the present study, NRPs are highly expressed in the human [22] and mouse bladder urothelium and intramural ganglia in close association with VEGF receptors [23]. Moreover, urothelial-related diseases and BCG-induced inflammation alter NRP expression and the accessibility of VEGF to these receptors [22,23]. In addition, NRPs also regulate neuronal plasticity as indicated by mutant mouse studies, showing that peripheral nerve regeneration is delayed in neuropilin 2-deficient mice [24]. The latter observation raises the question of whether upregulation of VEGF and guidance molecule expression during inflammation would also lead to altered nerve plasticity.

Little information is available regarding SEMA concentrations in non-malignant bladders. Semaphorins are a large family of signaling proteins that are both secreted and membrane bound. A common theme in the mechanisms of semaphorin function is that they alter the cytoskeleton and organization of actin filaments and the microtubule network [25]. Class 3 semaphorins (Sema3A-G) are the only secreted forms in vertebrates. Among the class 3 semaphorins, Sema3A has been most intensively studied in relation to axon guidance [26] and intrathecally administered Sema3A protein attenuates neuropathic pain behavior in rats with chronic constriction injury of the sciatic nerve [27]. Sema3A shows repulsive activity toward a variety of neuronal types [28]. Sema3A and its receptors (NRP1, NRP2, plexin A1, plexin A2, and plexin A3) were found to be significantly increased during M-CSF-mediated differentiation of monocytes into M2 macrophages of the inflammatory phenotype [29].

Relative to cross-talk among signaling molecules, it is interesting that VEGF and semaphorins have opposite effects on the filopodia of both endothelial cells (ECs) and axons expressing neuropilins. Sema3F, the semaphorin ligand of NRP2, is known to repel nerves [30] and endothelial cells [31], whereas VEGF_165 _attracts the filopodia, which drives the ECs or axons to move in the direction of the VEGF gradient [30].

For these studies, BCG was chosen to induce cystitis because it is known to: **a**) provoke a significant increase in VEGF expression in the urinary bladder [32], **b**) cause profound inflammation that is dependent on the VEGF pathway [33], **c**) up-regulate the urothelial expression of VEGF receptors and NRPs [23], and **d**) induce IL-17 up-regulation [33] and its receptors that are necessary for nerve regeneration [34].

The innovative results of this research provide evidence that chronic inflammation induces alterations of bladder peripheral nerve density that express: a) the transient receptor potential vanilloid subfamily 1 (TRPV1) [35], b) protein gene product (PGP9.5)[36], c) substance P, and d) calcitonin gene-related peptide (CGRP). Results obtained with potent and specifically engineered neutralizing antibodies against VEGF, NRP1, and NRP2 further suggest a putative mechanism underlying inflammation-induced increase in peripheral nerve density. Furthermore, instillation of VEGF into the bladder recapitulated the effects of BCG on inflammation and nerve plasticity.

## Results

### Chronic instillation of BCG, VEGF_165_, and VEGF_121 _result in bladder inflammation

**Figure 1:**
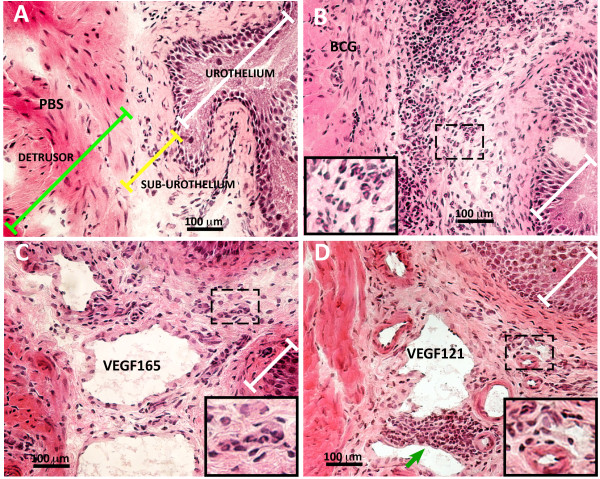
**Mouse bladder inflammation**. Representative photomicrographs from the urinary bladders isolated from mice chronically treated with 4 weekly instillations of PBS (**A**), BCG (**B**), VEGF_165 _(**C)**, and VEGF_121 _(**D**). Black box inserts on **B**, **C**, and **D **correspond to a 200% magnification of the original dashed box area of the respective figures and illustrate inflammatory cells. White transversal bars indicate the urothelium, yellow bars indicate the sub-urothelium, and green bars indicate the detrusor smooth muscle. Green arrow indicates perivascular infiltrate of inflammatory cells (**D**). See Figures 2 and 3 for quantification of inflammatory cells by image analysis.

As BCG [33] and VEGF_121 _[22] once instilled into the mouse bladder are absorbed across apparently intact urothelium and systemically distributed [22], we sought to compare the degree of inflammatory cell infiltrate in response to these stimuli. Because VEGF_165 _contains amino acid residues that bind to heparin and heparan sulfate proteoglycans, it is partly diffusible and partly bound to the pericellular matrix [12] which may explain its poor diffusion into deeper bladder layers. Therefore, we decided to also test the VEGF_121 _isoform that is freely diffusible [12], since it lacks the basic amino acid residues responsible for heparin binding and, therefore, does not, or only minimally, binds to the extracellular matrix (ECM). Figure 1 illustrates inflammation in response to chronic instillations of BCG, VEGF_165 _and VEGF_121_. This illustration shows a predominant cellular infiltrate in response to chronic BCG instillation, whereas VEGF_165 _and VEGF_121 _have a more profound impact in the bladder vascular system leading to dilation of both arteries and veins. Higher magnification inserts are amplification of dashed square areas of the respective figures and illustrate inflammatory cells and perivascular infiltrates (Figure 1).

**Figure 2:**
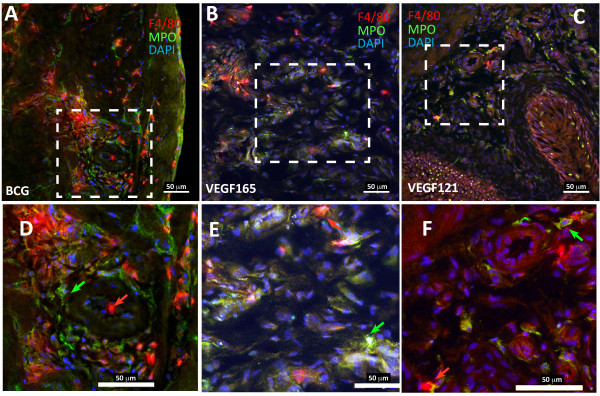
**Representative photomicrographs of double immunofluorescence (MPO and F4/80) of the urinary bladder mucosa isolated from mice that received 4 weekly instillations of BCG (A and D), VEGF_165 _(B and E), and VEGF_121 _(C and F)**. Qualitatively all stimuli induced the migration of both F4/80+ macrophages and MPO+ neutrophils. Quantification of the results is being present in Figures 3 and 4 and summarized on Table 1. D, E, and F correspond to magnification of the original dashed box area of the respective figures A, B, and C.

**Figure 3:**
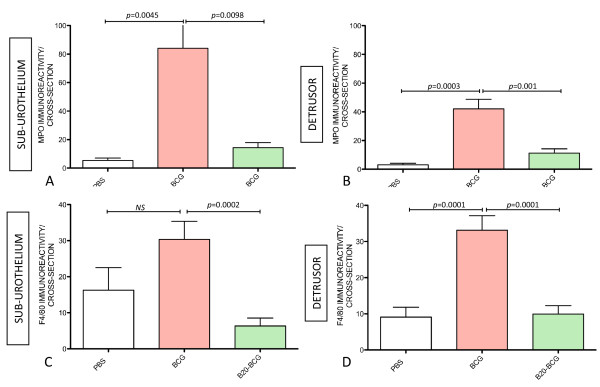
**Chronic BCG instillation induces a predominant migration of myeloperoxidase-positive neutrophils and, to a lesser extent, F4/80-positive macrophages that are blunted by co-treatment with VEGF neutralizing antibody (B20)**. Quantification of myeloperoxidase-IR (**A-B**) and F4/80-IR (**C-D**) in the sub urothelium and detrusor muscle of bladders isolated from: control (mice receiving i.p. PBS [twice a week] concomitant to 4 weekly instillations of PBS), BCG-treated (mice receiving i.p. PBS [twice a week] concomitant to 4 weekly instillations of BCG), and B20-treated (mice receiving i.p. B20 [5 mg/kg; twice a week] concomitant to 4 weekly instillations of BCG). N = 8 per group. Statistical significance was set for *p *values < 0.05.

Next, double immunofluorescence was used to further clarify whether the inflammatory infiltrate in response of BCG and VEGF was composed of MPO+ and F4/80+ cells (Figure 2). Quantitative analysis of MPO+ and F4/80+ cells indicate that chronic BCG instillation induces both neutrophils and macrophages infiltrations (Figure 3). In the sub-urothelium, BCG induced predominantly neutrophils (Figure 3A and 3B) that were almost double the number of macrophage (Figure 3C and 3D). These results are summarized on Table 1.

**Table 1:** Comparisons between BCG- and VEGF-induced inflammatory cell migration (MPO and F4/80), vascular response (CD31), and nerve plasticity (PGP9.5 and TRPV1) in the sub-urothelial layer of the mouse bladder.

%PBS	BCG 1 WK	VEGF165 1 WK	VEGF165 4 WKS	VEGF165 6 WKS	VEGF121 1 WK
**MPO**	1, 682 ± 442	107 ± 33	441 ± 91	363 ± 77	222 ± 44
**F4/80**	190 ± 31	252 ± 48	318 ± 40	398 ± 102	414 ± 58
**CD31**	163 ± 7	251 ± 22	144 ± 14	167 ± 25	220 ± 15
**PGP9.5**	414 ± 48	533 ± 74	257 ± 44	385 ± 67	151 ± 40
**TRPV1**	223 ± 14	205 ± 11	183 ± 20	146 ± 20	134 ± 17

**Figure 4:**
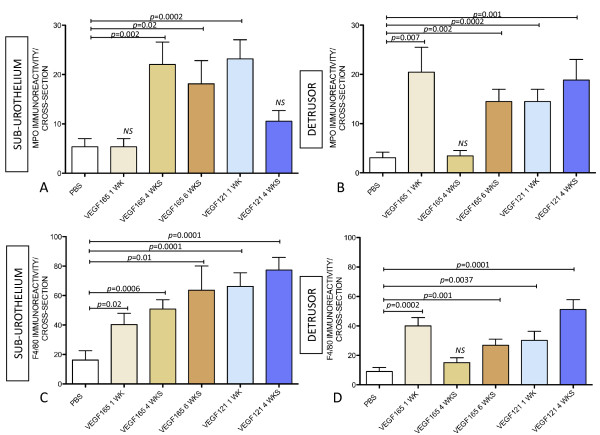
**VEGF induces bladder inflammation**. Quantification of myeloperoxidase-IR (**A-B**) and F4/80-IR (**C-D**) in the sub urothelium and detrusor muscle of bladders isolated from: 4 weekly instillations with PBS, single instillation with VEGF_165_, 4 weekly instillations with VEGF_165_, 6 weekly instillations with VEGF_165_, a single instillation with VEGF_121_, and 4 weekly instillations of VEGF_121_. Mice were euthanized one week after the last instillation and the urinary bladders removed for IF. N = 8 per group. Statistical significance was set for *p *values < 0.05.

Individual values illustrated on Figures 3, 4, 8, 9, 10, 11, and 13A were expressed as % of the respective PBS group and the average ± standard error of the mean are summarized.

As bladder instillation with BCG is known to provoke a significant increase in VEGF levels [32,33] concomitant to up-regulation of VEGF receptors and co-receptors (NRPs) [23], we tested whether BCG-induced inflammation was dependent on VEGF. To remove the influence of VEGF, an additional group of mice received systemic treatment with neutralizing VEGF antibody twice a week, concomitant to four weekly instillations of BCG. The results presented in Figure 3 indicate that neutralization of VEGF by B20 resulted in a blunted inflammatory response characterized by a reduction of both neutrophils and macrophages towards baseline levels. As these results suggest that VEGF participates in bladder inflammation, additional groups of mice received instillations of VEGF_165 _and VEGF_121_. Interestingly, both forms of VEGF induced a predominant infiltration of macrophages in the sub-urothelial layer (Figure 4C and table 1) and, to a lesser extent when compared to BCG (Figure 3A and Table 1), some neutrophil infiltration (Figure 4A and Table 1).

### BCG-induced inflammation results in bladder nerve plasticity

**Figure 5:**
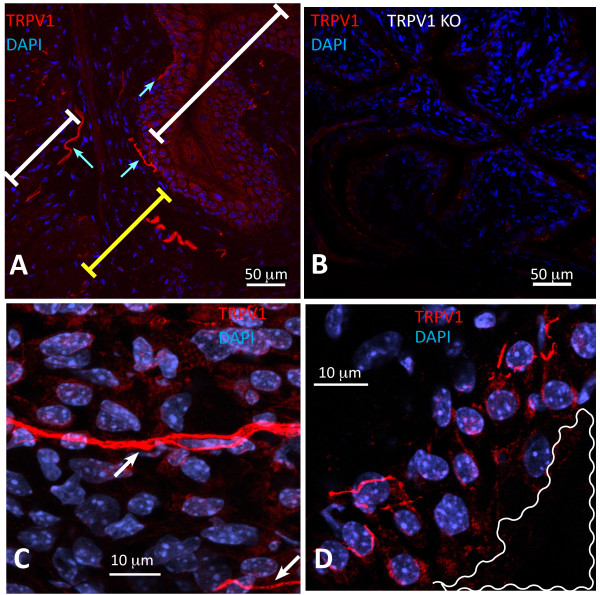
**TRPV1-IR in the mouse urinary bladder**. Representative photomicrograph illustrating distribution of TRPV1-IR in the urinary bladder isolated from C57BL/6 mice receiving chronic PBS instillation (**A**) shows TRPV1-IR extending from the base (cyan arrow) of the urothelium (white transversal bar) towards the sub-urothelium (yellow transversal bar). **B**) No immunostaining was observed in bladders isolated from TRPV1^-/- ^mice indicating that this antibody specifically identifies TRPV1-IR nerve fibers. **C **indicates some TRPV1-positive nerves crossing the urothelial layer (white arrows) and **D **illustrates TRPV1-positive nerves surrounding urothelial cells. White waved line on **D **highlights the bladder lumen.

**Figure 6:**
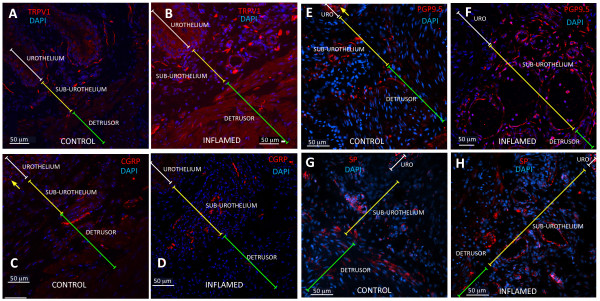
**Mouse bladder nerves**. Representative photomicrographs illustrating distribution of IR for: TRPV1 (**A-B**), CGRP (**C-D**), PGP9.5 (**E-F**), and SP (**G-H**) in urinary bladders isolated from C57BL6 mice that received chronic PBS instillation (**left panels**) or chronic BCG instillation (**right panels**). White transversal bars indicate the urothelium, yellow bars indicate the sub-urothelium, and green bars indicate the detrusor smooth muscle. Yellow arrows indicate non-specific labeling of urothelial cells with PGP9.5 (**F**) and CGRP (**C**). See Figure 6 for quantification of IR by image analysis.

**Figure 7:**
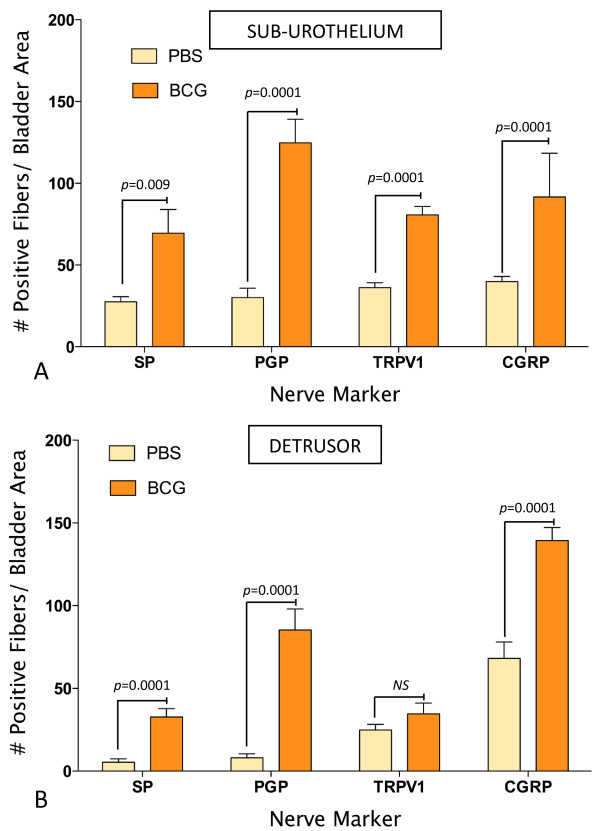
**Chronic BCG instillation increases IR for the pan neuronal marker, PGP9.5, and sensory nerve markers, TRPV1, SP, and CGRP**. Quantification of PGP9.5- TRVP1-, SP-, and CGRP-IR in the sub urothelium (**A**) and detrusor muscle (**B**) of bladders isolated from: control (mice receiving i.p. PBS [twice a week] concomitant to 4 weekly instillations of PBS) and BCG-inflamed mice (mice receiving i.p. PBS [twice a week] concomitant to 4 weekly instillations of BCG). N = 8 per group. Statistical significance was set for *p *values < 0.05. NS = non significant.

**Figure 8:**
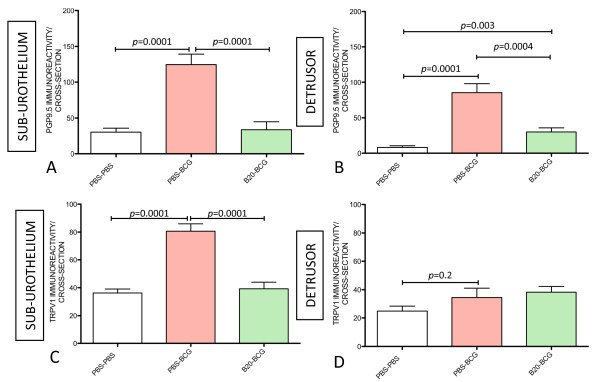
**VEGF mediates chronic BCG-induced bladder nerve plasticity**. Quantification of PGP9.5-IR (**A-B**) and TRPV1-IR (**C-D**) in the urinary bladder isolated from: control (mice receiving i.p. PBS [twice a week] concomitant to 4 weekly instillations of PBS), BCG-treated (mice receiving i.p. PBS [twice a week] concomitant to 4 weekly instillations of BCG), and B20-treated (mice receiving B20 [twice a week] concomitant to 4 weekly instillations of BCG). N = 8 per group. Statistical significance was set for *p *values < 0.05.

In order to determine whether chronic bladder inflammation results in alteration of nerve density, we first determined the distribution of sensory nerves expressing the nociceptive transducer vanilloid type 1 transient receptor potential receptor (TRPV1) in the urinary bladder isolated from control mice. The results illustrated in Figure 5A confirmed the reported distribution of TRPV1 immunoreactivity (IR) in the rat bladder [1] and extend the results to nerves crossing the urothelial layer (Figure 5C) or surrounding urothelial cells (Figure 5D). TRPV1 antibody specificity was verified by comparing the labeling of bladders isolated from C57BL/6 (Figure 5A) used as a positive control and TRPV1^-/- ^mice [37] used as a negative control (Figure 5B) to bladders from WT mice (TRPV1^+/+^; Figures 5C-D). No immunostaining was observed in bladders isolated from TRPV1^-/- ^mice (Figure 5B), indicating that this antibody specifically identifies TRPV1-IR in the urothelium, sub-urothelium, and detrusor nerve fibers (Figures 6A-B). Figures 6A and 6B are low magnification micrographs in order to illustrate the distribution of TRPV1-positive fibers in several layers of the urinary bladder isolated from control (PBS) and inflamed bladders (BCG), respectively. Regardless of magnification, Figures 6A-B illustrate that inflammation induced a higher degree of TRPV1-IR in the sub-urothelium of bladders isolated from BCG-treated mice that was confirmed by image analysis (Figure 7). Next, we compared TRPV1-IR with other nerve markers. For this purpose, tissues isolated from the same groups were also stained with antibodies identifying CGRP (Figures 6C-D), PGP9.5 (Figures 5E-F), and SP (Figures 6G-H). It was noted that PGP9.5 and CGRP antibodies resulted in a non-specific labeling of the urothelial layer. A reasonable explanation for this artifact is that the methods recommended for permeabilization of the tissues caused such labeling. These findings precluded the use of the urothelium for quantification purposes, and image analysis was performed in two layers: the detrusor smooth muscle and the sub-urothelium that extended from the basal layer of the urothelium to detrusor. Results obtained with image analysis indicate that BCG induced a significant increase in the pan-neuronal marker PGP9.5-IR in the sub-urothelium and detrusor smooth muscle (Figure 8) and an increase in sensory nerve marker TRPV1-IR, specifically in the sub-urothelium (Figure 8). BCG also induced a significant increase in CGRP- and SP-IR (Figure 7).

### VEGF mediates BCG-induced alterations in bladder nerve plasticity

It was reported that VEGF is expressed in nerves at relatively higher amounts than in the surrounding mesenchymal tissue [8], and a new appreciation of the role of VEGF in neuronal development emerged [12,38] that made us review a possible link between BCG-induced inflammation and bladder nerve plasticity with the activity of VEGF. As neutralization of VEGF by B20 resulted in a blunted inflammatory response to BCG (Figure 3), next we investigated whether BCG-induced alteration in nerve plasticity was also modulated by VEGF. Tissues obtained from mice that received PBS, BCG, or BCG and B20 were submitted to image analysis to quantify PGP9.5- and TRPV1-IR in the sub-urothelium and detrusor smooth muscle. The results presented in Figures 8 indicate that neutralization of VEGF by B20 completely blocked the capacity of BCG to increase PGP9.5 (Figures 8A-B) and TRPV1-IR (Figures 8C-D) suggesting that the observed increase in nerve plasticity was mediated by VEGF acting either directly or indirectly on the nerves. Control experiments included treatment of mice with Avastin that recognizes and neutralizes human VEGF but is inactive against mouse VEGF. Avastin did not alter the enhanced innervation of the urinary bladder following instillation of BCG in mice (data not shown).

### Blocking NRP antibodies

**Figure 9:**
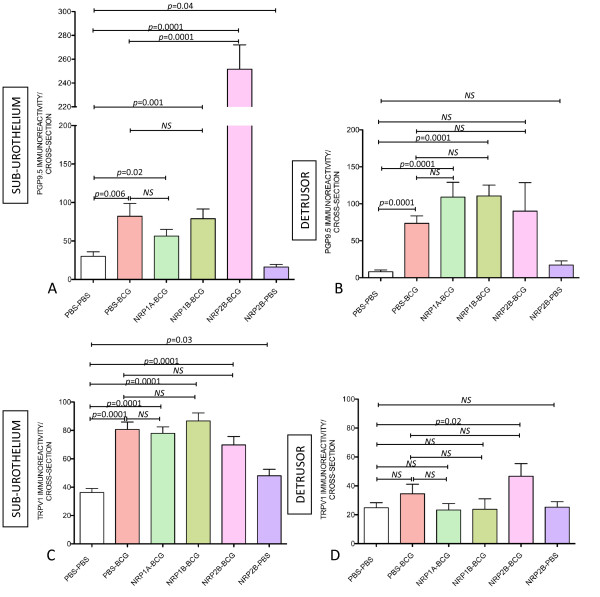
**Effect of NRP1 and NRP2 blocking antibodies on BCG-induced bladder nerve plasticity**. Quantification of PGP9.5-IR (**A-B**) and TRPV1-IR (**C-D**) in the urinary bladder isolated from: control (mice receiving i.p. PBS [twice a week] concomitantly with 4 weekly instillations of PBS), BCG-treated (mice receiving i.p. PBS [twice a week] concomitant to 4 weekly instillations of BCG), NRP1^A^-BCG treated (mice receiving NRP1^A ^[twice a week] concomitant to 4 weekly instillations of BCG), NRP1^B^-BCG treated (mice receiving NRP1^B ^[twice a week] concomitant to 4 weekly instillations of BCG), NRP2-BCG treated (mice receiving NRP2^B ^[twice a week] concomitant to 4 weekly instillations of BCG), and NRP2-PBS treated (mice receiving NRP2^B ^[twice a week] concomitant to 4 weekly instillations of PBS). N = 8 per group. Statistical significance was set for *p *values < 0.05.

To further explore the mechanism involved in VEGF mediated inflammation and nerve plasticity, we used NRP antibodies since blockade of Neuropilin-VEGFR coupling is significantly more effective than other approaches in decreasing VEGF-VEGFR2 signaling [39,40]. Based on the capacity of NRP antibodies to significantly reduce lymphatic vessel proliferation and neutrophil migration during chronic bladder inflammation induced by BCG [33], we tested whether the blockade of NRP1 or NRP2 with engineered antibodies would also alter the effect of BCG on bladder nerve density. Figures 9A-D indicate that concomitant treatment with NRP1^A ^or NRP1^B ^did not change the capacity of BCG to induce an increase of TRPV1-IR or PGP9.5-IR. These results suggest that NRP1 may not be involved in BCG-induced bladder neuronal plasticity. Surprisingly, blockade of NRP2 with a specific antibody (NRP2^B^) resulted in strong potentiation of BCG effects on sub-urothelial PGP9.5-IR (Figure 9A). Regarding TRPV1-IR, NRP2^B ^did not further enhance the substantial increase in sensory nerves induced by BCG in the sub-urothelium (Figure 9C). However, the combination of BCG and NRP2^B ^treatments resulted in a significant increase of TRPV1-IR in the detrusor muscle (Figure 9D).

### Substance P and CGRP-IR positive nerves are also increased by the combination of BCG and NRP2^B^

**Figure 10:**
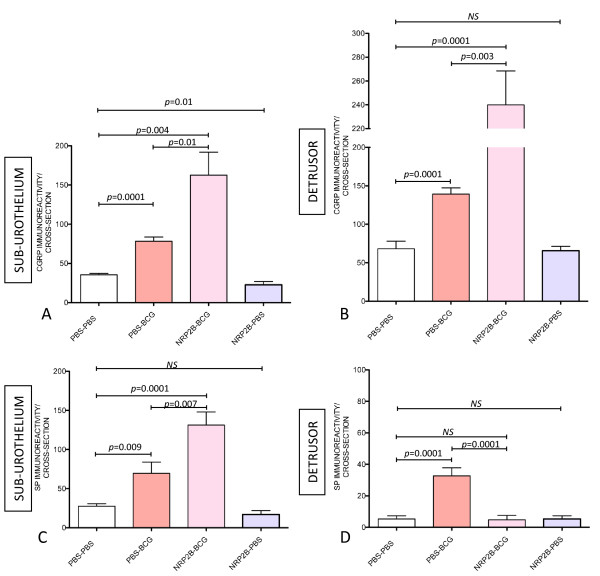
**Effect of NRP2 blocking antibodies on BCG-induced CGRP- and SP-IR**. Quantification of CGRP-IR (**A-B**) and SP-IR (**C-D**) in the urinary bladder isolated from: control (mice receiving i.p. PBS [twice a week] concomitant to 4 weekly instillations of PBS), BCG-treated (mice receiving i.p. PBS [twice a week] concomitantly with 4 weekly instillations of BCG), and NRP2-BCG treated (mice receiving NRP2^B ^[twice a week] concomitant to 4 weekly instillations of BCG). N = 8 per group. Statistical significance was set for *p *values < 0.05.

To further explore the effects of combined therapy of NRP2^B ^and BCG, we tested whether increased nerve plasticity was specific for TRPV1-IR or a general effect on sensory nerves expressing other markers such as SP and CGRP. Combined treatment of NRP2^B ^and BCG induced a significant increase of CGRP-IR in the sub-urothelium (Figure 10A) and detrusor muscle (Figure 10B) and substance P-IR in the sub-urothelium (Figure 10C).

### VEGF instillation induces bladder nerve plasticity

**Figure 11:**
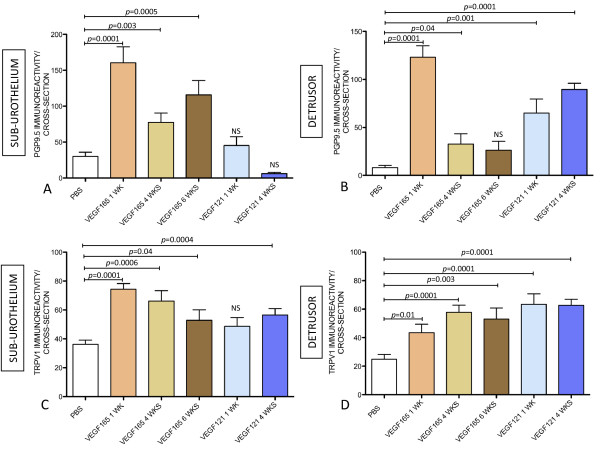
**VEGF_165 _and VEGF_121 _recapitulate BCG-induced bladder nerve plasticity**. Quantification of PGP9.5-IR (**A-B**) and TRPV1-IR (**C-D**) in the urinary bladder isolated 1 week after: 4 weekly instillations with PBS, single instillation with VEGF_165_, 4 weekly instillations with VEGF_165_, 6 weekly instillations with VEGF_165_, a single instillation with VEGF_121_, and 4 weekly instillations of VEGF_121_. N = 8 per group. Statistical significance was set for *p *values < 0.05.

To provide direct evidence that VEGF induces bladder neuronal plasticity, VEGF_165 _and VEGF_121 _were instilled into the mouse bladder either acutely (1 WK = single instillation of VEGF and mice were euthanized one week after) or chronically (4 WKs = four weekly instillations of VEGF and tissues removed 1 week after the last instillation). In order to determine if the bladder responses to VEGF_165 _have reached a plateau, an additional group received six weekly instillations of VEGF_165 _and was euthanized 1 week after the last instillation. One week after a single instillation of VEGF_165 _into the mouse bladder, a substantial increase in the number of PGP9.5-IR and TRPV1-IR fibers was observed, and this increase was at least double the basal number of nerves in the sub-urothelium and detrusor smooth muscle (Figure 11). After the 4^th ^weekly treatment with VEGF_165_, a tempering of the response was observed. In addition to VEGF_165_, VEGF_121 _significantly increases PGP9.5-IR in nerves running into the detrusor smooth muscle (Figure 11B) and TRPV1 fibers in the sub-urothelium (Figure 11C) and detrusor smooth muscle (Figure 11D). Table 1 summarizes these results.

### Differences between bladder responses to BCG and VEGF

**Figure 12:**
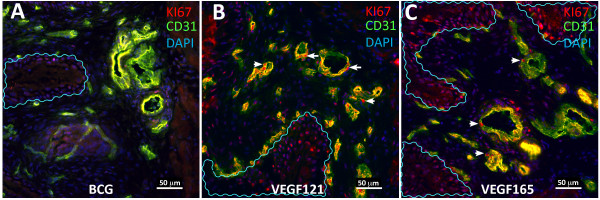
**Representative photomicrographs of double immunofluorescence (CD31 and KI67) of the urinary bladder mucosa isolated from mice instilled chronically (4 weekly instillations) of: BCG, VEGF_165_, and VEGF_121_**. The area of CD31+ blood vessels was used to calculated microvessel density (Figure 13A) and the co-localization between CD31+ endothelial cells and KI67 expression was used to calculate the degree of angiogenesis (Figure 13 B).

**Figure 13:**
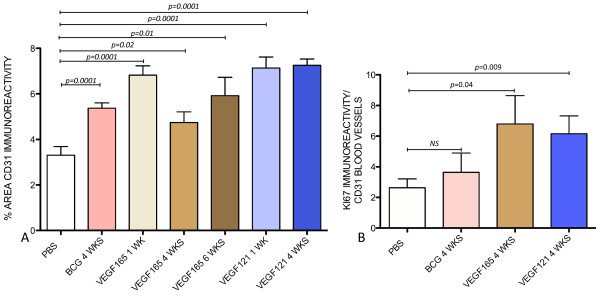
**Effects of instillation of BCG, VEGF_165_, and VEGF_121 _into the urinary bladder resulted in alterations of vascular plasticity**. (A) Microvessel density (MVD) was determined by computing the area occupied by CD31+ blood vessels that was expressed as percent of the cross-sectional area. (B) Angiogenesis was defined by the proliferation of CD31+ blood vessels. For this purpose, the expression of a marker of proliferation (KI67) was calculated taking CD31+ blood vessels as region of interest (ROI).

The effects of chronic instillation of BCG, VEGF_121_, or VEGF_165 _into the mouse bladder on microvessel density and cell proliferation were conducted by determining the area of CD31-positive blood vessels an the expression of KI67. Figure 12 contains representative photomicrographs of CD31 positive microvessels along with the expression of KI67. In tissues obtained from BCG-treated mice, KI67 expression was predominantly observed on urothelial cells (waved blue lines on Figure 12) and to a lesser extend in CD31+ blood vessels (Figure 12A) whereas in tissues isolated from VEGF_121_- (Figure 12B) and VEGF_165_-treated mice (Figure 12C), in addition to urothelial cells, a strong expression of KI67 occurred on CD31+ blood vessels. Figure 13A compare the CD31+ microvessel density on tissues stimulated with BCG or VEGF. Figure 13B presents the quantification of KI67 taking the CD31+ cells as region of interest and indicates that in contrast to BCG, chronic VEGF instillation into the mouse bladder induced angiogenesis.

## Discussion

This manuscript presents a body of evidence implicating VEGF signaling in the enhanced innervation of the urinary bladders following instillation of BCG in mice. Qualitatively these results add to a list of evidence implicating the VEGF pathway in the bladder responses to BCG. A quantitative analysis of inflammatory infiltrate and vascular plasticity indicates possible alternative pathways activated by BCG and VEGF.

The nervous and vascular systems share several anatomical parallels. Both systems utilize a complex branching network of neuronal cells or blood vessels reaching all regions of the body. The anatomical similarity of the nervous and vascular systems suggests that axons might guide blood vessels and vice versa [30]. Indeed, signal molecules produced by peripheral neuronal cells, such as VEGF [8], guide blood vessels [12], and signals from vessels, such as the neurotrophins NGF and NT-3, are required for, and orchestrate extension of neurons adjacent to vessels [41]. In this manner, the neuronal and vascular systems are well organized and coordinated in normal adult tissues. However, in chronic inflammatory states particularly in the LUT, little is known about how the nerve-vessel relationship functions and whether it could underlie the chronic pain syndrome observed in certain patients.

Interest in guidance molecules, and particularly VEGF, modulating both vascular and neuronal pathology is emerging. VEGF levels are associated with alterations in the bladder vascular system. VEGF is increased in bladders of patients with painful bladder syndrome, and it is associated with glomerulations on hydrodistension [42]. However, increased bladder VEGF is not observed in patients who failed to show petechial bleeding or in controls [42]. In addition, VEGFR-1 and NRP2 expressions are reduced in bladder biopsies from patients with cystitis [22].

We reasoned that pro-inflammatory stimuli known to increase VEGF, and in particular NRP expression, might also modulate inflammation-induced nerve plasticity. The contribution of chronic inflammation to peripheral nerve plasticity was investigated in a murine model using BCG instillations that it is known to increase signaling molecules, such as IL-17 [34] and VEGF [23], that are necessary for inducing neuronal plasticity. Our results confirmed our hypothesis that intravesical instillation of BCG increased the density of PGP9.5-, SP-, TRPV1-, and CGRP-IR within the bladder wall. For some parameters, the numbers of sensory nerves identified closely parallels numbers of PGP9.5 immunoreactive nerves, which (accepting the limitations of sensitivity of staining techniques) could indicate that most PGP9.5 fibers observed were peptidergic sensory nerves. However, further studies on sympathetic nerve staining and non-peptidergic nerve afferents are necessary to determine the whole extend of BCG- and VEGF-induced neuronal plasticity.

A limitation of our experimental conditions is that concentrations of neutralizing antibodies and the pro-inflammatory stimuli were probably different among the different layers of the urinary bladder. The antibodies were systemically administered, whereas pro-inflammatory stimuli were instilled into the bladder. Therefore, we analyzed the effects on both the sub-urothelium and detrusor muscle, and the results obtained seem to reflect differences in tissues that are in closer proximity to the stimulus. Interestingly, BCG induced an increase of TRPV1-IR in the sub-urothelium but not in the detrusor smooth muscle, whereas PGP9.5-IR was found increased in both layers. As BCG favors an increase of inflammatory cells primarily in the sub-urothelial layer when compared to the detrusor muscle, these results suggest TRPV1-IR, but not PGP9.5-IR, is associated with the degree of inflammatory infiltrate. In this context, in the absence of inflammatory cell infiltrate, such as in IL-17 knockout mice, a significant decrease in mechanical pain hypersensitivity was observed [43]. In contrast, when neutrophil infiltration was increased by administration of IL-17, a concomitant increase in neuropathic pain was observed [43]. Furthermore, depletion of circulating neutrophils at the time of nerve injury significantly attenuated the induction of hyperalgesia [44].

Besides neutrophils and macrophages that were quantified in the present study, other inflammatory cells should be considered to have an action in neural plasticity. In particular, NRPs are recognized as a new marker for regulatory T (Treg) cells [45,46] and are expressed in antigen presenting cells and effector cells [47,48]. In addition to NRPs, SEMAs seem to participate in inflammation as it has been suggested that macrophages and fibroblasts secrete SEMAs that may be responsible for reduced sympathetic innervation [49]. Therefore, it is fair to propose that one possible mechanism by which chronic BCG instillation induces bladder neuronal plasticity is by attracting inflammatory cells that will contribute to increased tissue levels of VEGF or SEMA.

At this time, there is not definitive evidence correlating the type of inflammatory cell involved in the regulation of bladder sensory nerve plasticity. However, given the known trophic effects of VEGF on neurite growth [34,50] prolonged survival of neurons [51,52], and re-enervation following local nerve damage [53,54], it is fair to propose that inflammatory cells producing VEGF may be involved in the observed neural plasticity. This new appreciation of VEGF signaling in bladder inflammation is supported by emerging evidence that levels of various VEGF subtypes are, in general, increased at the site of inflammation, and that infiltrating lymphocytes and other inflammatory cells represent an additional source of VEGF [55].

The present work introduces intriguing results regarding NRPs. As NRP1 has a domain structure strikingly similar to that of NRP2 [18], we first tried to disrupt binding of VEGF and SEMA to NRP1 using blocking antibodies that were engineered to block either the a1-a2 domain or b1-b2 domains of NRP1. Both antibodies, although proven to reduce BCG-induced inflammation, angiogenesis, and vascular remodeling [56], failed to alter the effects of BCG on nerve density. This suggests that either the antibodies are specific for impairing inflammation-induced angiogenesis or that NRP1 may not participate in mechanisms underlying neuronal plasticity in the urinary bladder. An alternative explanation is based on findings that spatial gradients of Sema3A and VEGF may promote differential NRP1 binding [50]. Indeed, vessels expressing high levels of Sema3A favor NRP1-PlexinA1 signaling, producing chemorepulsive cues limiting sympathetic neurite outgrowth and vascular enervation, while low Sema3A expressing vessels favor NRP1-VEGFR2 signaling that provides chemoattractive cues for sympathetic neurite outgrowth and vascular enervation [50].

The unexpected increase in nerve density after NRP2^B ^administration deserves further investigation and studies are underway in our laboratory to define the mechanism/s involved in bladder responses to this antibody. It is also interesting that NRP2^B ^alone did not induce alteration of nerve density. However, when given concomitantly with BCG, NRP2^B ^induced an overwhelming increase in bladder nerve fibers. One possible explanation is that NRP2^B ^might potentiate nerve plasticity by activating angiogenesis or increasing the migration of inflammatory cells such as neutrophils and macrophages. Our preliminary results indicate that NRP2^B ^alone does not alter bladder blood vessel density or the number of inflammatory cells (data not shown). However, NRP2^B ^does increase both F4/80+ macrophages and MPO+ neutrophils in response to BCG (data not shown).

It seems that in addition to activate the VEGF signaling pathway, BCG may have induced an increase in SEMAs. This would explain the finding that NRP2^B ^further increased the number of nerves when administered concomitant to BCG. As SEMAs are known for strong chemorepulsion, these results suggest that blockade of NRP2 reduces SEMA participation and the consequent repulsion of neurons. Although NRP2^B ^antibody was originally developed to target the coagulation V/VII factor (b1-b2) domains of NRP2 which are required for VEGF-C binding to NRPs [21], and the b1-b2 domains do not directly engage SEMA, this antibody could decrease SEMA binding by preventing NRP dimerization that is necessary for accommodating SEMA domains that pack tightly together at this interface [57].

It is possible that other secreted semaphorins binding to NRP2 alone are responsible for the chemorepulsion and the consequent effect of NRP2^B ^antibody on neural plasticity. NRP2 is known to be required for mediating repulsive actions of Sema3B, 3C, and 3F, whereas NRP1 is known to be required for Sema3A function. In fact, Sema3B and Sema3F seem to require only NRP2, not NRP1, to elicit their effects, whereas Sema3C may require both NRP1 and NRP2 [58]. Another piece of evidence indicating that NRP2 may play a role in altered nerve plasticity is the finding that peripheral nerve regeneration is delayed in NRP2-deficient mice [24], indicating that this guidance molecule facilitates peripheral-nerve axonal regeneration.

We also found evidence that target-derived VEGF_165 _or VEGF_121 _plays a previously unrecognized role in promoting growth of bladder nerves and inflammation. A role for VEGF in inflammation has been postulated. However, instillation of VEGF into the mouse bladder represents direct evidence that VEGF induces inflammation and that this new animal model can be used to investigate the effects of elevated levels of VEGF on bladder neuronal and vascular plasticity.

A fundamental question raised by the present findings is whether the increased innervation occurred as a consequence of angiogenesis or inflammation. It is known that innervation typically accompanies blood vessels [10] which made it important to determine whether the observed effects on nerves are direct, or mediated through effects on angiogenesis. This was a difficult topic to address facing the evidence indicating an overlap between inflammation and angiogenesis. We found that increased microvessel density (MVD) was part of the bladder responses to chronic instillations of BCG, VEGF_165_, and VEGF_121_. However, BCG did not induce a significant endothelial cell proliferations as indicated by KI67 and therefore, the increased MVD observed in response to BCG was probably related to the intense vasodilation known to be part of the inflammatory response. In contrast, proliferating endothelial cells were only observed in VEGF-treated tissues and, therefore, it seems that angiogenesis underlies the bladder responses to VEGF, as indicated by an increased KI67 expression on CD31+ endothelial cells [59].

Although an increase in nerve density, particularly those expressing TRPV1-IR, has been proposed to underline pain sensation and neurogenic detrusor overactivity based on findings that desensitization of afferents with capsaicin and resiniferatoxin decrease pain and detrusor instability [60], the present work did not explore whether the increased nerve density corresponded to an increased function of the sensory system. The primary goal of the present work was to explore putative mechanisms involved in inflammation-induced neural plasticity before conducting a detailed study on nerve function. Our results were focused on the target organ, and future studies should include the consequences of VEGF instillation on more central neurons such as those of the dorsal root ganglia. In this context, it should be recognized that while plasticity of the central nervous system (in response to stimuli) and regeneration (in response to injury) are mainly based on adaptive changes in neural circuits and synaptic reorganization, plasticity of the peripheral nervous system is predominantly based on axonal re-growth and neuron addition [61].

## Conclusions

VEGF is not only involved in angiogenesis, but also in an inflammatory reaction and neuronal plasticity. Chronic BCG administration into the mouse urinary bladder resulted in pronounced inflammation accompanied by increased density of PGP9.5, TRPV1, SP, and CGRP nerves. BCG effects on inflammatory cells and nerves were nullified by neutralizing VEGF antibody and qualitatively reproduced by intravesical administration of VEGF_165 _or VEGF_121_. However, VEGF-induced neural plasticity seems to associate with angiogenesis whereas BCG-induced neural plasticity seems to be a consequence of inflammation. Concomitant administration of the VEGF-inducer, BCG, along with neutralizing anti-NRP2 antibodies resulted in a surprising potentiation of neuronal plasticity, whereas NRP1 antibodies did not. The present work suggests the novel concept that both inflammation and angiogenesis can lead to increased bladder nerve plasticity.

## Methods

### Experimental Bladder Inflammation

All animal experimentation conformed to the APS's Guiding Principles in the Care and Use of Animals and was approved by the OUHSC Animal Care & Use Committee (protocol #08-105). Urinary bladders isolated from TRPV1^-/- ^(B6.129S4- *Trpv1^tm1Jul^*/J; Jackson Laboratory) and age-matched control mice (TRPV1 ^+/+^; WT) were generously provided by Dr. Avelino (Faculty of Medicine of Porto) and used for confirmation of TRPV1 antibody specificity. For all other studies, ten- to 12-week old C57BL/6 female mice (Jackson Laboratory, Bar Harbor, ME) were used. Female mice (n = 8 per group) were anesthetized with ketamine HCl (40 mg/kg i.m.) and xylazine (2.5 mg/kg i.m.), transurethrally catheterized with a polypropylene catheter (24 gauge; ¾ in.; Angiocath, Becton-Dickinson, Sandy, UT), and test compounds were instilled at a slow rate to avoid trauma and vesicoureteral reflux, 150 μl of one of the following substances were instilled: pyrogen-free saline (PBS; controls), BCG (TheraCys-Aventis-Pasteur; total dose of 1.35 mg [33], mouse recombinant VEGF_165 _(15 nM), or VEGF_121 _(15 nM). To ensure consistent contact of substances with the bladder and to avoid reflux or leakage, the catheter was occluded and left in place for 30 minutes. Groups of mice were euthanized 24 hours post instillation, and the urinary bladders were removed for histology (H&E staining) and immunofluorescence studies. The concentrations of BCG, VEGF_165_, and VEGF_121 _used in this study were selected because they induce bladder inflammation when instilled into the mouse bladder.

### Systemic administration of neutralizing antibodies

The following blocking antibodies were administered systemically to C57BL/6 female mice (n = 8 per group): B20, anti-NRP1^A^, anti-NRP1^B^, and anti-NRP2^B^. Synthetic phage antibody libraries were used by Genentech, incorporation to produce the synthetic blocking antibodies: B20, NRP1A, NRP1B, and NRP2B. The libraries were generated using a single human framework with a template containing human consensus complementarity-determining regions (CDRs) [62] and the antibodies that cross-react with both human and murine NRP were generated. B20 was isolated from synthetic antibody phage libraries [62,63], and this neutralizing antibody binds and blocks both human and murine VEGF [63]. Anti-NRP1^A ^was developed to neutralize the a1-a2 domain of NRP1 responsible for binding semaphoring [64], and anti-NRP1^B ^neutralizes the b1-b2 domain responsible for binding of VEGF [64]. Anti-NRP2^B ^was developed to specifically target the coagulation V/VII factor (b1-b2) domains of NRP2 responsible for the binding of VEGF_165 _and VEGF-C and, thereby decreasing lymphangiogenesis [21]. Mice were randomly assigned to one of the following groups that received intraperitoneal injections of 150 μl of: PBS, B20 (5 mg/kg), control antibody (Avastin, bevacizumab, 5 mg/kg [64]), anti-NRP1^A ^[10 mg/kg [64], anti-NRP1^B ^[10 mg/kg [64], anti-NRP2^B ^(40 mg/kg). Mice were treated on *day 0*, and then twice a week for 5 weeks. Mice concomitantly received weekly intravesical instillations of 150 μl of PBS or BCG (TheraCys; total dose 1.35 mg, Sanofi-Pasteur [33]) for 4 consecutive weeks.

### Immunofluorescence (IF) of mouse tissues

Urinary bladders were processed for IF according to published methods [56] to determine the effects of BCG and VEGF on the density of nerves. Briefly, mice were exposed to isofluorane, and when anesthesia was obtained, mice were euthanized by cervical dislocation, bladders were removed, urine was drained, and tissues were frozen immediately in 1:1 tissue freezing media (TFM, Triangle Biomed Sciences) and OCT (Tissue Tek^®^). For all tissues, appropriate cross-section morphology was confirmed by H&E staining and examination by light microscopy prior to preparing slides for IF labeling. Frozen sections (10 μm thick) were post-fixed in 1% MeOH-free formaldehyde for all staining except for the CGRP antibody as subsequently described. Briefly, slides were blocked for 35 minutes with 5% normal donkey serum (NDS; Jackson Immunolabs), then co-incubated with primary antibodies in 0.5% NDS for 90 minutes in a humidified chamber or overnight at 4°C. When double IF was used, following brief rinses with PBS, slides were co-incubated with both secondary antibodies at the same time. All secondary antibodies were used at a 1:400 dilution and included donkey anti-rabbit IgG Alexafluor 488 and 546 conjugate (Molecular Probes), donkey anti-goat IgG Alexafluor 546, donkey anti-rat IgG Alexafluor 488, and goat anti-guinea pig 546. Slides were washed, counterstained with 4', 6-diamidino-2-phenylindole (DAPI), and coverslipped. Controls included slides labeled only with individual primary and secondary antibodies, as well as secondary antibodies only. CGRP labeling was performed by fixing tissues with 4% p-formaldehyde for 90 minutes at 4°C, then blocking with 10% NDS/0.3% Triton/0.1% BSA for 1 hour at room temperature. Slides were incubated with the CGRP antibody in PBS/0.3% Triton/0.1% BSA overnight at 4°C in a humidified chamber. After brief washes the slides were stained with the appropriate secondary antibody and counterstained with DAPI and coverslipped.

### Primary antibodies

TRPV1 antibody (1:10, 000) was raised in rabbits against the 15 C-terminal amino acids of the rat TRPV1 sequence [37]. Commercially available antibodies included: rabbit anti-human protein gene product 9.5 [PGP9.5] (Neuromics; catalog # RB12103, 1:1500 dilution), rabbit anti-mouse substance P (Millipore; catalog AB1566; 1:250 dilution), guinea pig anti-rat substance P (Millipore; catalog AB15810; 1:1000 dilution), rabbit anti-rat CGRP (Immunostar; catalog # 24112; 1:1000 dilution), rabbit anti-human myeloperoxidase (Dako; catalog #A0398; 1:600 dilution), rat anti-mouse Pecam (CD31; BD Pharmingen; catalog 550274; 1:50 dilution), goat anti-mouse KI-67 (Santa Cruz; catalog # sc-7846; 1:75 dilution), and goat anti-mouse F4/80 (Santa Cruz; catalog# sc-26642; 1:100 dilution).

### Image analysis

Sections were viewed and photographed using a Zeiss LSM-510META scanning confocal microscope (Thornwood, NY) using a Zeiss axiocam HRm high-resolution CCD camera, driven by Axiovision 4.6 software. For image analysis, all tissue cross-sections were viewed with a Nikon Eclipse TE 2000-S inverted fluorescent microscope and imaged at room temperature using a digital CCD camera (Roper Scientific; Sarasota, Florida 34240) driven by NIS-Elements AR 3.0 Imaging software. DAPI staining was viewed using a DAPI filter set (340-380 nm ex, 435-485 nm em). Imaging of Alexafluor 488 utilized an excitation filter of 465-495 nm and an emission filter of 515-555 nm. Alexafluor 546 was imaged with an excitation of 528-553 nm and 590-650 nm emission range. A control slide stained only with secondary antibody was used to determine exposure time and to set minimum background fluorescence levels for each fluorophore imaged. Once set, exposure times were not changed during acquisition of each respective fluorophore in the staining series. Staining was considered positive only when the acquired signal exceeded the established background. Absence of signal bleed-through was determined using previously optimized multi-acquisition settings on single fluorophore stained slides.

### Inflammatory cells

For image analysis of MPO+ and F4/80+ positive inflammatory cells, NIS-Elements AR 3.0 Imaging software was set to include signals with an equivalent diameter between 1-10.9 μm^2 ^[The equivalent diameter is a size feature derived from the area. It determines the diameter of a circle with the same area as the measured object: Eqdia = sqrt (4*Area/p)] and to exclude any signal with an area smaller than 1 μm^2^.

### Nerve fibers

Histologically nerve fibers undulate in and out of the plane of the section, sometimes appearing as linear structures, and sometimes punctate staining, presumed indicating nerves in cross-section. Therefore, the following parameters were used in order to exclude structures above or below a certain size as being potentially non-neuronal and to exclude inflammatory cells since monocytes and macrophages have been reported to express TRPV1 [65]. In this context, for image analysis of nerves, the NIS-Elements AR 3.0 Imaging software was set to count only structures with length between 0.19-500 μm and width 0.19-2.5 μm. [Length is a derived feature appropriate for elongated or thin structures. Length = (Perimeter + sqrt (Perimeter^2 ^- 16*Area))/4]; and Width is a derived feature appropriate for elongated or thin structures. It is based on the rod model and is calculated according to the following formula: Width = Area/Length].

### Blood vessels and Angiogenesis

Microvessel density (MVD) was determined by computing the area occupied by CD31+ blood vessels that was expressed as percent of the cross-sectional area. Angiogenesis was defined by the proliferation of CD31+ blood vessels. For this purpose, the expression a marker of proliferation (KI67) was calculated taking CD31+ blood vessels as region of interest (ROI).

To meet the independent randomized sampling assumption required for our statistical test(s), the following measures were taken: blinding the reader to treatment groups and picking a random starting position and proceeding clockwise with 6-10 non-overlapping images. As 12-20 fields are necessary to view the whole bladder cross-section at 400× magnification, the sampling of 6-10 non-overlapping images represented half of the entire bladder cross-section. The area occupied by cells stained positively by a particular antibody was calculated as percent of the total area of the region of interest (ROI), as indicated in the individual figure legend and the results of 6-10 fields were averaged. This procedure was repeated for all 8 bladders used per treatment group and the results are expressed as mean ± SEM of 8 cross-sections. The data were examined to determine if the distributions were homoscedastic and Gaussian. As these conditions were met, groups were compared through a two-sample Student's t test. An alpha of 0.05 was considered statistically significant. *P*-values were adjusted for multiple comparisons through a Bonferroni correction.

This manuscript reports the results obtained with 13 experimental groups (PBS i.p. and 4 weekly instillations of BCG; PBS i.p. and 4 weekly instillations PBS; single instillation of VEGF_165_, 4 weekly instillations of VEGF_165_, 6 weekly instillations of VEGF_165_; single instillation of VEGF_121_; 4 weekly instillations of VEGF_121_; avastin i.p. and 4 weekly instillations of BCG; B20 i.p. and 4 weekly instillations of BCG; NRP1^A ^i.p. 4 weekly instillations of BCG; NRP1^B ^i.p. 4 weekly instillations of BCG; NRP^2B ^i.p 4 weekly instillations of BCG; and NRP2^B ^i.p. 4 weekly instillations of PBS), containing 8 mice per group, for a total of 104 mice. In addition to H&E, image analysis was performed in least 6-10 non-overlapping images of all bladder cross-sections stained with 8 different antibodies (CD31, Ki67, PGP, TRPV1, CGRP, SP, MPO, and F4/80). The rationale for performing *in vitro *image analysis of nerves, blood vessels, and inflammatory cells using the same cross-sections obtained from animals submitted to 13 different treatments was to permit the proper comparisons. In order to better organize the results and to improve the manuscript readability the results were separated in different sections. The latter does not imply that experiments were performed in addition to the ones described above.

### Reagents

Mouse recombinant VEGF_165 _(catalog # MGC70609) and VEGF_121 _(catalog # CYT-574) were purchased from ProSpec-Tany TechnoGene Ltd (Rehovot 76124, Israel). TheraCys^© ^BCG was purchased from Sanofi-Pasteur. Anti-NRP1^A^, anti-NRP1^B^, anti-NRP2^B^, B20, and Avastin were obtained from Genentech Corp.

## Competing interests

The authors declare that they have no competing interests.

## Authors' contributions

All authors read and approved the final manuscript. **MRS **designed the study, performed the *in vivo *experiments, and drafted the manuscript, **CAD **performed IF imaging and analysis, **AA **participated in the experimental design, provided TRPV1 tissues and antibody, **FC **participated in the experimental design, **JM **performed tissue section, advised with IF techniques, and helped **CAD **with confocal photomicrographs, **DEB **participated in the experimental design and helped draft the manuscript, **TJS **participate in the experimental design, interpretation of results, and draft of the manuscript, **REH **participated in the experimental design, **RS **conceived the study.
